# Degraded EEG decoding of wrist movements in absence of kinaesthetic feedback

**DOI:** 10.1002/hbm.22653

**Published:** 2014-10-12

**Authors:** Ferran Galán, Mark R. Baker, Kai Alter, Stuart N. Baker

**Affiliations:** ^1^ Institute of Neuroscience, Newcastle University Newcastle upon Tyne, NE2 4HH United Kingdom

**Keywords:** afferent feedback, deafferentation, motor imagery, motor commands, brain–machine interface, electroencephalography

## Abstract

A major assumption of brain–machine interface research is that patients with disconnected neural pathways can still volitionally recall precise motor commands that could be decoded for naturalistic prosthetic control. However, the disconnected condition of these patients also blocks kinaesthetic feedback from the periphery, which has been shown to regulate centrally generated output responsible for accurate motor control. Here, we tested how well motor commands are generated in the absence of kinaesthetic feedback by decoding hand movements from human scalp electroencephalography in three conditions: unimpaired movement, imagined movement, and movement attempted during temporary disconnection of peripheral afferent and efferent nerves by ischemic nerve block. Our results suggest that the recall of cortical motor commands is impoverished in the absence of kinaesthetic feedback, challenging the possibility of precise naturalistic cortical prosthetic control. *Hum Brain Mapp 36:643–654, 2015*. © **2014 The Authors. Human Brain Mapping Published by Wiley Periodicals, Inc**.

## INTRODUCTION

Brain–machine interface (BMI) technology offered early promise of restoring independence to those with spinal cord injury. Grounded in seminal work from awake behaving monkey [Evarts, 1968; Georgopoulos et al., 1982], BMI research aims to decode movement parameters from neural ensemble activity, enabling natural control of assistive devices. However, the speed and accuracy of current BMIs [Collinger et al., 2013; Hochberg et al., 2012] are poor compared to natural movements and highly dependent on visual feedback, showing some similarities with motor deficits in patients with sensory neuropathies. This observation led us to speculate that absent [Collinger et al., 2013; Hochberg et al., 2012] and arbitrary (O'Doherty et al., 2011] sensory feedback produced by controlling an artificial actuator (e.g., robotic arm) interferes with the recruitment of the neural population previously engaged in controlling a natural effector with intact feedback. Rather than viewing the spatiotemporal sequence of activity which produces movement as internally generated by cortical circuits in a feedforward manner [Churchland et al., 2012; Shenoy et al., 2013], such a view would extend the “dynamical machine” [Shenoy et al., 2013] responsible for movement to include afferent feedback from the periphery. This is supported by evidence for the rapid integration of sensory feedback into motor output, while taking account of high‐level movement goals [Krutky et al., 2010; Pruszynski et al., 2011]. In such a framework, loss of feedback would have an impact comparable to the lesion of a cortical area. Movement might still be possible, but only after reconfiguration of the network, and is likely to be impoverished compared with the natural state. It is known that primary sensory and motor areas undergo plastic changes [Brasil‐Neto et al., 1993; Cohen et al., 1991; Merzenich et al., 1983; Sanes et al., 1988] associated with abnormal function [Cramer et al., 2005] when afferent inputs are removed. Moreover, studies with amputees suggest that access to the motor representation of the missing limb is conditional upon the re‐establishment of peripheral connections and restoration of the sensorimotor loop [Reilly et al., 2006], an observation also supported by experiments in patients with hand allografts [Vargas et al., 2009] and targeted muscle reinnervation for prosthetic control [Kuiken et al., 2007].

**Figure 1:**
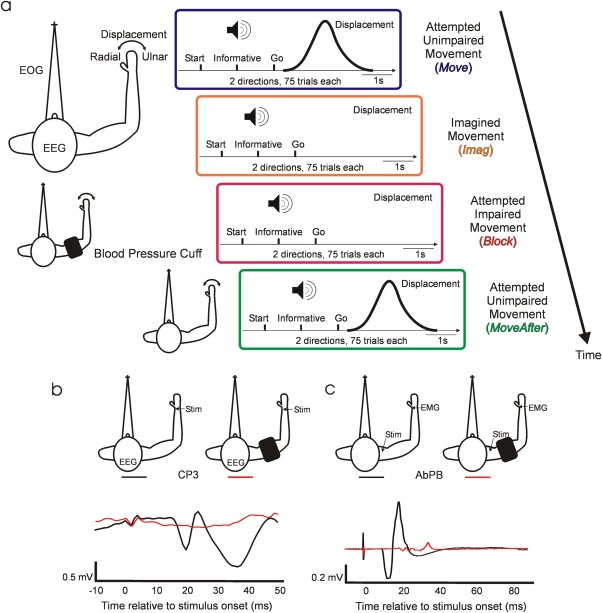
Experimental setup. (**a**) Behavioral task. Diagrams depict the temporal structure within trials and between conditions. Auditory cues indicate trial start, instructed deviation, and “Go.” Schematic views of the arm, from above, describe subjects' position and experimental setup during task performance. The top subject schematic, corresponding to *Move* and *Imag*, displays the electrophysiological signals and displacement measured during all experimental conditions. The middle subject schematic illustrates the position of the blood pressure cuff during *Block* condition. The bottom subject schematic corresponds to *MoveAfter*. Only conditions *Move* and *MoveAfter* involve movements. (**b**) Afferent block. Example of averaged SEP recorded at CP3 following median nerve stimulation before (black) and after (red) deafferentation by the ischemia. After deafferentation N20 is absent. (**c**) Efferent block. Example of CMAP recorded at muscle abductor pollicis brevis following supramaximal Erb's point single pulse magnetic stimulation before (black) and after (red) motor block.

We hypothesized that cortical motor commands cannot be effectively generated in the absence of kinaesthetic feedback. Here, we tested this hypothesis, using temporary ischemic nerve block to model disconnection of cortical circuits from the periphery. In the same subjects, we compared electroencephalography (EEG) decoding of unimpaired movements (*Move*/*MoveAfter*) with the same movements attempted during peripheral disconnection (*Block;* see Fig. 1a). We further evaluated decoding of imagined movements (*Imag*), which also lack movement reafference and have been used to calibrate decoders for people with tetraplegia [Hochberg et al., 2006, 2012] (Fig. 1a,b). We found that effective decoding was only possible when displacement‐triggered reafference was present, suggesting that cortical motor commands deteriorate when they cannot be updated by their sensory consequences. This challenges the possibility of precise naturalistic cortical prosthetic control in patient groups with peripheral disconnection.

## MATERIALS AND METHODS

### Experimental Setup

Nine healthy adults (7 men, 2 women) took part in the study. All the experimental procedures were approved by the Research Ethics Committee of the Medical Faculty, Newcastle University; subjects provided written informed consent to participate. Each subject performed four experimental conditions in the following order: unimpaired movement (*Move*), imagined movement (*Imag*), attempted movement after ischemic nerve block (*Block*) and unimpaired movement after circulation had been restored to the arm (*MoveAfter*) (see Fig. 1a). Subjects were instructed to sit comfortably in a chair fixating on a static visual target placed approximately 150 cm ahead to equalize visual feedback across conditions. The right elbow and pronated forearm were gently placed at waist height onto an arm rest, which restricted movement to radial and ulnar deviation at the wrist joint (lateral hand movements). The hand was placed flat with extended fingers on the rest, which was padded with memory foam. The same foam imprints were used across sessions to minimize postural changes. The rest was custom made to fit each subject's arm well, minimizing hand movements and movement around other joints. Wrist angular displacement was sensed by a potentiometer, fixed with its axis coaxial to the wrist joint. A displacement of 0° indicated the neutral position with the hand in the same plane as the forearm; positive angles denoted radial deviation. The left arm rested unrestrained in a comfortable position throughout the task. Each trial commenced with an auditory start cue (2,000 Hz; 100 ms), followed 1 s later by an informative cue (100 ms) indicating the required direction on that trial: radial (1,500 Hz) or ulnar (500 Hz) deviation. After a 1s delay period a “Go” cue (1,000 Hz; 100ms) indicated when to initiate the movement. In *Move* and *MoveAfter* conditions, subjects were instructed to perform fast, stereotyped radial/ulnar deviations of the wrist. In the *Imag* condition, subjects were requested to imagine radial/ulnar deviations as performed during the immediately preceding *Move* trials but without overt movement. In *Block* condition, subjects were instructed to try to perform the movements as in *Move*, despite the impairment produced by nerve block. Each condition consisted of a randomized sequence of 150 trials (75 in each direction) and lasted 13 min.

### Data Acquisition

Scalp EEG was recorded by a 61‐sensor cap according to the International 10–20 system referenced to Cz. Electro‐oculograms (EOG) were recorded via bipolar electrodes; horizontal EOG was recorded by placing an electrode to the outer canthus of each eye, and vertical EOG by an electrode pair above and below the subjects' left eye. Both EEG and EOG signals were sampled at 1 kHz (Neuroscan SynAmps 2RT, Compumedics USA, Charlotte, NC) and grounded with an electrode placed over the left clavicle. Impedance for all electrodes was <5 kΩ. To record stimulus‐evoked responses (see below), bipolar surface electromyogram was recorded from the right abductor pollicis brevis (AbPB), amplified and high‐pass filtered at 30 Hz (D360, Digitimer, Welwyn, UK), and sampled at 5 kHz (CED Micro1401, Cambridge, UK). A ground electrode was placed on the dorsum of the wrist.

### Ischemic Nerve Block Procedure

Ischemic nerve block was achieved by applying a commercial blood pressure cuff to the right arm at the level of the biceps. The arm was first raised for ∼30s to drain blood from the large veins; the cuff was then rapidly inflated to a pressure of 180 mm Hg. The arm was then gently lowered and placed into the apparatus. Cuff pressure was maintained constant through this experimental condition. Subjects were told not to contract muscles distal to the cuff, from the moment that the arm was raised, until the experimental recording began. As we emphasized the importance of this instruction, subjects were able to remain relaxed throughout, and thereby avoided muscle pain associated with lactate buildup.

Somatosensory evoked potentials (SEPs) following electrical stimulation of the median nerve at the wrist (stimulus rate 9 Hz, pulse width 1 ms, intensity just below motor threshold, 1,000 stimuli) and compound muscle action potentials (CMAPs) of the AbPB muscle following magnetic nerve stimulation (Magstim 200, Dyfed, UK; single pulse; intensity supramaximal) at the supraclavicular fossa (Erb's point) were monitored to assess sensory and motor block. Baseline SEPs and CMAPs were measured before applying the cuff. Beginning 18 min after cuff inflation, SEPs were measured at intervals of 2 min until the contralateral N20 was absent, indicating complete block of large fiber sensory afferents (see Fig. 1b). At this stage the subjects no longer perceived the nerve stimulus. Two minutes after this point, CMAPs were measured at intervals of 30 s until their amplitude was significantly reduced, indicating almost complete motor block (see Fig. 1c). Subjects were then requested to attempt to perform the task (*Block* condition). Immediately after task completion (75 trials in each direction) the cuff was removed, limiting the maximum total ischemic time to 50 min. The *MoveAfter* condition started 5–10 min after cuff removal, when subjects no longer reported reperfusion paresthesias.

### Data Analysis

#### Signal processing

EEG and EOG single trial epochs were extracted from 500 ms before to 2,500 ms after the informative cue. In *Move* and *MoveAfter* conditions, trials with no movement, movement in the wrong direction, or movement with a reaction time five times the median absolute deviation above the mean were excluded. Previous studies have shown that low‐frequency components carry significant information about hand movement direction [Mehring et al., 2003; Rickert et al., 2005; Waldert et al., 2008; Wang et al., 2010] allowing for close‐loop inference of wrist movements [Witte et al., 2014]; therefore, data were filtered to include only these frequencies (1–40 Hz) and rereferenced (common average reference [CAR]). Poor quality EEG channels were excluded before computing CAR. Time‐resolved amplitudes of oscillations in the 1–40 Hz frequency range were computed using complex Morlet wavelets (2 s time resolution at 1 Hz central frequency). EOG data were low‐pass filtered with a cut off at 30 Hz (second‐order Butterworth, zero phase shifts).

#### Cortical sources of EEG surface activity

**Figure 2:**
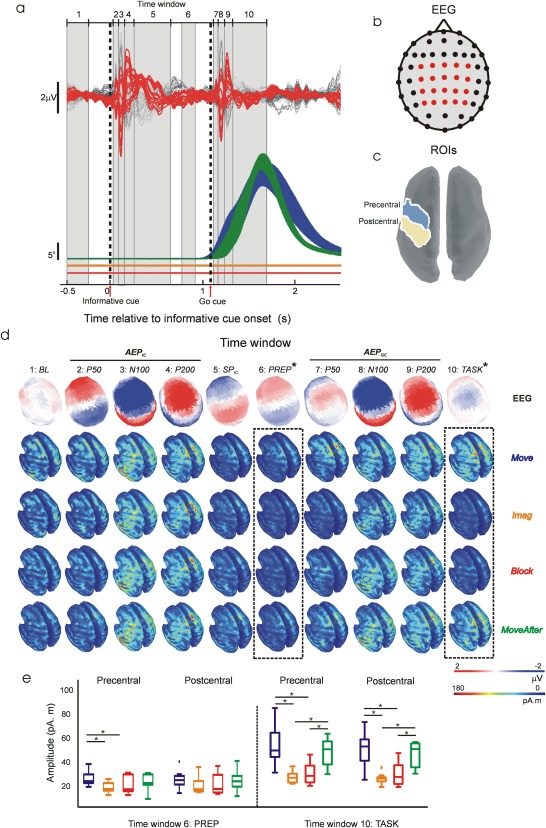
Temporal dynamics of EEG source activity. (**a**) Top, EEG grand averages across subjects. In red, traces corresponding to channels included in decoding analysis. Bottom, averaged (±SEM) absolute displacement across subjects. Color codes condition as in Figure 1a. Displacement traces for *Imag* and *Block* conditions have been shifted downwards slightly for clarity. Vertical dashed lines represent “informative” and “Go” cue onsets. Grey numbered boxes define the 10 windows used for analysing the dynamics of EEG source activity (see Materials and Methods). (**b**) EEG channels' topographic distribution. Red channels correspond to red traces in a. (**c**) Localization of precentral and postcentral ROIs used for analysing EEG source activity. (**d**) Grand averages across subjects of time averaged EEG potentials' topographic distribution and absolute cortical source activity within each time window defined in a. Dashed line boxes indicate windows in which significant differences in cortical source activity were found between conditions. (**e**) Box plots of averaged source activity at precentral and postcentral ROIs from each subject in time windows that revealed significant differences between conditions (windows 6 and 10 highlighted with dashed 2line boxes in d). * In d and e denotes significant difference (*P* < 0.01).

Cortical sources of single‐trial EEG surface activity were estimated by computing Tikhonov‐regularized minimum‐norm estimates [Baillet et al., 2001] on a symmetric Boundary Element Method (BEM) head model [Gramfort et al., 2010] using constrained dipoles (15,000 vertices normal to cortical surface) and standard Tikhonov regularization ( λ=0.1). The cortical current maps were analyzed using regions of interest (ROIs) defined by Tzourio‐Mazoyer atlas onto Colin 27 volume coordinates [Holmes et al., 1998]. ROI activity was computed as the averaged absolute activity of all the vertices included in the ROI. Temporal dynamics of ROI activity was examined by measuring mean source activation over time samples included within 10 time windows spanning relevant trial periods identifiable in EEG and displacement grand averages (see Fig. 2a,d): −500 to −250 ms before informative cue onset (window 1; baseline, BL), 15–60 ms (window 2; auditory evoked potential in response to informative cue, *AEP_IC_ P60*), 60–120 ms (window 3; *AEP_IC_ N100*), 120–240 ms (window 4; *AEP_IC_ P200*), 240–600 ms (window 5; slow potential after informative cue *SP*_IC_), 750–900 ms (window 6; task preparation, *Prep*), 1,115–1,165 ms (window 7; AEP by “Go” cue, *AEP_GC_ P60*), 1,165–1,220 ms (window 8; *AEP_GC_ N100*), 1,220–1,300 ms (window 9; *AEP_GC_ P200*), and 1,330–1,700 ms after informative cue onset (window 10; task execution*, Task*).

Spatial goodness of fit of the estimated sources at the time bin of maximum DA was estimated with the averaged coefficient of determination (*R* ^2^) across trials and subjects for each condition.

#### Decoding wrist deviation from electrophysiological signals

**Figure 3:**
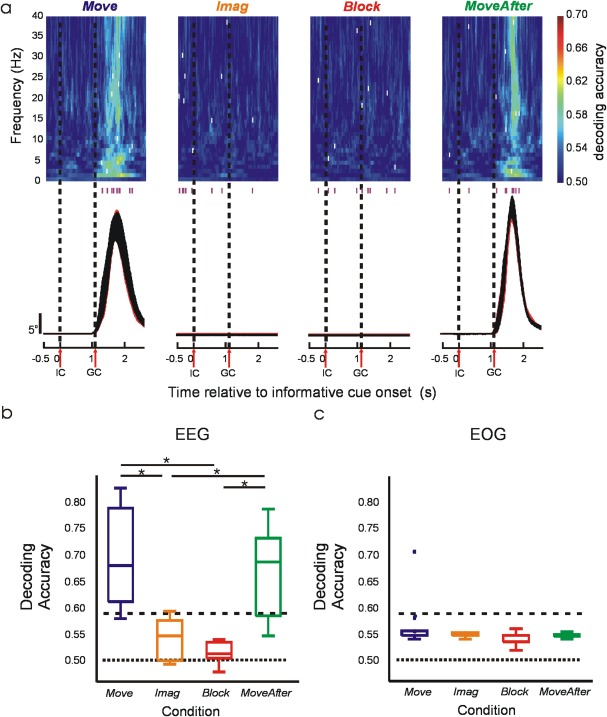
Decoding of wrist deviation. (**a**) Top, subject‐averaged decoding accuracy (DA) in time‐frequency space using EEG signals. White ticks within the color map indicate time‐frequency bins with maximum DA from each subject; the time of these bins is also shown with purple ticks beneath the color map. Bottom, average absolute displacement (±SEM) across subjects. Vertical dashed lines represent “informative” and “Go” cue onsets. (**b**) Box plots of DA from each subject using EEG signals. (**c**) Box plots of maximum DA from each subject using EOG signals. Horizontal dotted and dashed lines represent chance level of 50% and the value above which DA deviates significantly (*P* < 0.05) from the chance level. *Significant pairwise difference (*P* < 0.01).

Wrist deviations were decoded using a Bayes linear classifier (see classifier description below). Decoding accuracy in Figure 3a was estimated for each condition by leave‐one‐out cross‐validation across 40 frequency components and 3,000 time bins (corresponding to 1–40 Hz range and 3 s epochs sampled at 1 kHz) using the estimated amplitudes from 20 EEG channels covering bilateral sensorimotor areas (FC3, FC1, FCz, FC2, FC4, C4, C2, Cz, C1, C3, CP3, CP1, CPz, CP2, CP4, P4, P2, Pz, P1, P3). Note that decoding accuracy could almost certainly be increased by combining time‐frequency bins. However, our goal in this study was not to generate maximal decoding via an optimally tuned algorithm, but rather to compare EEG movement‐related information across experimental conditions. For this purpose, using single time‐frequency bins provided an assumption‐free comparison of information content at high resolution. Decoding accuracy in Figure 3b was estimated for each condition by leave‐one‐out cross‐validation, combining the arithmetic mean [Alexandre et al., 2001] of nine classifiers using the most informative time‐frequency bin from each subject (nine bins indicated as white ticks in Fig. 3a). To assess whether wrist movement direction could be inferred from correlated eye movements, decoding accuracy was estimated for each condition by leave‐one‐out cross‐validation across 3,000 time bins (3 s epochs sampled at 1 kHz) using vertical and horizontal filtered EOG.

#### Linear classifier

A Bayes linear classifier [Fukunaga, 1972] was used to decode wrist deviation d⊂J={radial,ulnar} from a signal vector of *N =* 20 EEG or *N =* 2 EOG. The likelihood functions were modeled as multivariate Gaussian distributions according to

(1) $$p(s|d)=\frac{1}{\sqrt{{\left(2\pi \right)}^{N}|C|}}e^{-\frac{1}{2}{\left(s-\mu_{d}\right)}^{T}C^{-1}(s-\mu_{d})},$$

where *s* depicts either the 20 dimensional signal vector comprising the amplitudes of a single frequency component recorded from 20 EEG channels at a certain time bin, or the 2‐dimensional signal vector comprising both filtered vertical and horizontal EOG at a certain time bin. *C* is the common, that is, deviation independent, covariance matrix and $\mu_{d_{}^{}}$ the deviation specific mean signal vectors.

For classification, the posterior probabilities were computed using Bayes' rule

(2) $$p(d|s)=\frac{p(d)p(s|d)}{\sum_{j\subset J}^{}p(j)p(s|j)},$$

where a uniform prior with p(d)=0.5 was used. Vector *s* was finally assigned to the deviation with the highest posterior probability.

#### Statistical analysis

Results for each condition are reported within the text as mean ± standard error of the mean (SEM). Within figures, the central line in box‐plots is the median, the box is defined by 25th and 75th percentiles, the whiskers extend to the most extreme data points not considered outliers, and outliers are plotted individually (∼ ±2.7σ). Differences between the four experimental conditions were examined using Friedman's test. Significance levels were corrected for multiple comparisons with a family‐wise error rate α=0.05. Significance of the decoding accuracy was examined with the cumulative binomial distribution [Mehring et al., 2003] using the lowest total number of trials across subjects ( n=140) to obtain a statistically conservative significance.

All analysis was performed offline in the MATLAB environment (The MathsWorks, Natick, MA). EEG filtering, EEG rereferencing, EEG time‐frequency decomposition and EEG source estimation were performed with Brainstorm [Tadel et al., 2011], which is documented and freely available for download online under the GNU general public license (http://neuroimage.usc.edu/brainstorm).

## RESULTS

Grand‐average EEG potentials and displacement traces are presented in Figure 2a. Informative and “Go” cues elicited the frontocentral P50‐N100‐P200 AEP complex and slower late potentials [Golob et al., 2002; Wood and Wolpaw, 1982]. No movement displacement was observed during *Imag* and *Block*. We defined 10 time windows of interest based on grand‐average EEG potentials and displacement traces to encompass relevant trial periods to our task (see Materials and Methods and Fig. 2a,d). Mean topographic distributions of grand average EEG potentials and condition mean source absolute activations over windows of interest are displayed in Figure 2d. Grand‐average EEG potentials during *AEP_GC_ P50* and *TASK* showed different topographies from *AEP_IC_ P50* and *SP_IC_* respectively indicating overlap with movement‐related potentials as suggested by displacement traces and source activations (Fig. 2a,d). EEG scalp potentials were mostly generated by temporal and sensorimotor sources partially overlapping in time as expected in an auditory‐motor delayed task (Fig. 2d); however, disturbances due to altered kinaesthetic feedback are expected in cortical areas responsible for the generation of motor commands during task preparation and/or execution (from window 6–10; *PREP*: *TASK*), rather than periods with no involvement of motor task (from window 1–5; *BL*: *SP_IC_*). Source analysis indeed revealed significant differences between conditions in the dynamics of sensorimotor sources generating movement‐related EEG scalp potentials (Fig. 2a,b). We defined ROIs to encompass precentral and postcentral cortex based on standard Atlas coordinates (Tzourio‐Mazoyer), and measured mean source absolute activation over these regions (Fig. 2c,d). Activity in the precentral ROI was significantly increased (*P* < 0.01) in *Move* condition compared with *Imag* and *Block* during movement preparation (*PREP*), and significantly increased (*P* < 0.01) in *Move* and *MoveAfter* compared with *Imag* and *Block* during maximum displacement (*TASK*); for a postcentral ROI, significant differences were also seen in *Move* and *MoveAfter* compared with *Imag* and *Block* during *TASK*. Neither precentral nor postcentral ROI activity differed significantly between conditions during periods with no involvement of motor task (from window 1–5; *BL*: *SP_IC_*) or during periods dominated by AEP*_GC_* (see Fig. 2d–e).

The activity of the precentral sources responsible for generating motor output was diminished in the absence of kinaesthetic feedback; however, this does not necessarily mean that the underlying motor commands which this reflected were impaired. To probe this in more detail, we performed a decoding analysis which attempted to predict the movement direction based on the EEG. We reasoned that if the smaller signals seem in *Imag* and *Block* conditions were still capable of good movement decoding, this would indicate some motor command nevertheless remained intact. In *Move* and *MoveAfter* conditions decoding performed significantly better (69.4 ± 3.1% and 66.3 ± 2.9%, respectively, *P* < 0.01) than by chance, but this was not the case for *Block* and *Imag* (51.6 ± 0.6% and 54.2 ± 1.3%, respectively, significantly lower than *Move* and *MoveAfter*, *P* < 0.01). Importantly, the plots of time‐resolved decoding accuracy (Fig. 3a) revealed that peak accuracy in the *Move* or *MoveAfter* conditions was temporally confined around task performance (see tick marks on and below color maps in Fig. 3a), as expected for a genuine neural signal. By contrast, peak decoding in *Imag* or *Block* conditions was distributed randomly over the analyzed timeframe, suggesting chance decoding of noise fluctuations unrelated to underlying neural processes.

**Figure 4:**
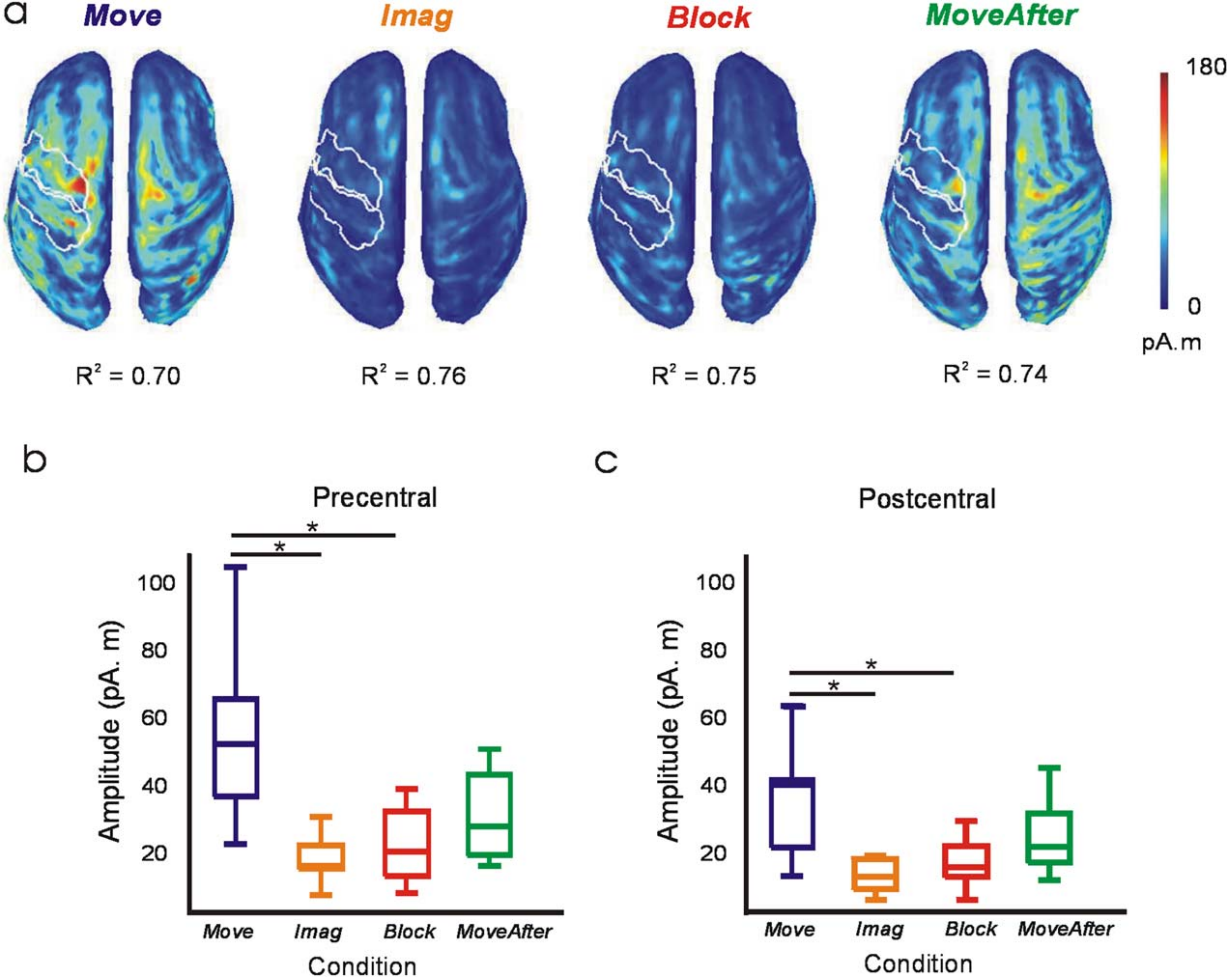
Cortical source activity during maximum DA. (**a**) Subject‐averaged absolute value of cortical source activity at time bins of maximum DA (see white ticks in Fig. 3a). Spatial goodness of fit of source activity in each condition is indicated by averaged *R* ^2^ values. White outlines define contralateral precentral and postcentral ROIs. (**b**, **c**) Box plots of averaged source activity at precentral and postcentral ROIs from each subject. *Significant pairwise difference (*P* < 0.01).

Further evidence of a key difference in the nature of the signals decoded came from source analysis at the time sample of maximum decoding accuracy (Fig. 4a). As expected, in the *Move* condition the contralateral primary sensorimotor areas were the major contributors of EEG surface activity. Activity in the precentral and postcentral ROI was significantly reduced (*P* < 0.01) in *Imag* and *Block* conditions compared with *Move* (Fig. 4b). Activity in *MoveAfter* seemed to restore partially, although not completely, back to that seen in *Move* (see Figs. 2e, 3b, 4b). Whilst in *Move* a strongly lateralized pattern was seen, the reduced activity contralaterally in *MoveAfter* led to a more balanced, bilateral cortical activation.

One possible confound with EEG decoding is that scalp potentials could be subtly influenced by eye movement artifacts. To check for this, we performed a similar decoding analysis as above, but using EOG signals (Fig. 3c). No significant decoding occurred in any condition, indicating that eye movements were uncorrelated with the instructed wrist movements and that EOG contamination of the EEG could not explain our results.

## DISCUSSION

This study shows that movement‐related information contained in EEG is impoverished in the absence of kinaesthetic feedback.

Previous work has correlated various movement parameters with motor cortical activity [Bradberry et al., 2009, 2010; Evarts, 1968; Georgopoulos et al., 1982; Jerbi et al., 2007; Kalaska et al., 1989; Mehring et al., 2003; Pistohl et al., 2008; Rickert et al., 2005; Schalk et al., 2007; Thach, 1978; Waldert et al., 2008; Witte et al., 2014], establishing the scientific basis for developing BMIs [Carmena et al., 2003; Ethier et al., 2012; Moritz et al., 2008; Serruya et al., 2002; Taylor et al., 2002; Velliste et al., 2008] that could confer intuitive neuroprosthetic control to patients suffering from paralysis [Collinger et al., 2013; Hochberg et al., 2012]. However, experiments with patients suffering large fiber sensory neuropathies [Ghez et al., 1995; Gordon et al., 1995; Rothwell et al., 1982; Sanes et al., 1984] have shown that without visual feedback the motor output of these patients fluctuates randomly. Visual feedback can only partially compensate for kinaesthetic loss in these patients, with movements becoming slow, inaccurate and requiring constant attention [Cole, 1995]. Kinaesthetic feedback plays an important role in motor control by allowing for error‐correction and by contributing in the formation of accurate internal models of limb dynamics [Wolpert et al., 1995; Wolpert and Miall, 1996]. These observations pose a relevant question to BMI research that, to our knowledge, has been unaddressed: to what extend does missing kinesthesia prevent the generation of the normal sequence of motor commands required for voluntary movements?

Here, we addressed this question by decoding from EEG activity attempted movements impaired by nerve block and imagined movements that lack movement reafference. Both conditions are relevant to our goal, but have their limitations. Ischemic nerve block provides a well‐established reversible model of short‐term amputation‐induced cortical reorganization in humans [Brasil‐Neto et al., 1993; Reilly et al., 2006] and provides a valid model for the loss of feedback from large diameter sensory fibers. However, the rapid onset of feedback loss, including the tonic level of drive seen in the steady state, could produce acute changes in cortical function not directly related to loss of movement‐related feedback. In addition, even over these short timescales, some cortical plasticity may occur; it was notable that the pattern of activation during maximum decoding was more bilateral in *MoveAfter* compared with *Move*. Such rapid and reversible cortical reorganization probably relies on the modulation of existent intracortical connections by afferent input [Ridding and Rothwell, 1999], potentially through a decrease of GABA‐mediated inhibition [Chen et al., 1998].

Conversely, imagined movements lack movement reafference, but preserve tonic feedback about the (unchanging) limb state. In imagined movements, central mechanisms presumably are also acting to prevent overt motor outflow [Guillot et al., 2012]. Importantly, imagined movements have been used to calibrate decoders for people with tetraplegia [Hochberg et al., 2006, 2012]. Using both of these noninvasive techniques in healthy subjects we were able to compare, for the first time, decoding of unimpaired, impaired and imagined movements. This overcomes some of the limitations presented by experiments with paralyzed patients and nonhuman primates: paralyzed patients are unable to perform goal‐directed movements (an essential condition to which all others should ideally be compared); by contrast, it is virtually impossible to control for nontask related movements and to assess the quality of motor imagery in nonhuman primates.

Our study revealed that the lack of reafferent feedback in imagined and impaired movements had an impact on the dynamics of the cortical sources generating movement related EEG scalp potentials. This appeared as reduced precentral cortical activity during preparation, and reduced precentral and postcentral cortical activity during execution of movements. Three further observations support the notion that cortical activity became grossly abnormal in the absence of sensory feedback. First, decoding accuracy dropped to chance levels. Second, the time of maximal decoding accuracy was no longer temporally locked around the time of attempted movement. Third, precentral and postcentral cortical activity at the time of maximum decoding was substantially reduced. Our findings extend to deafferentation those by other studies reporting reduced activity [Miller et al., 2010; Wang et al., 2010] and reduced decoding or tuning (Pandarinath, 2013; Wang et al., 2010] when comparing imagined with unimpaired movements, highlighting the critical role of kinaesthetic feedback for the successful recruitment of the neural population responsible for precise movement control.

Our study used EEG to access the neural signals underlying motor commands, and showed that decoding efficiency was reduced in the absence of a normal sensorimotor loop. We cannot exclude the possibility that motor processing continued relatively intact in these tasks, but that only the overt manifestation as discriminable scalp potentials was degraded. If so, this would imply that only EEG‐based naturalistic cortical prosthetic control will be challenged in paralyzed patients lacking feedback, whereas BMIs relying on invasively recorded single unit activity may still operate effectively. However, several pieces of evidence argue that our findings may also have applicability to invasive BMI. Pandarinath [Pandarinath, 2013] has reported weakened movement‐related multiunit modulation in human primary motor cortex (M1) during imagined movements, demonstrating that such degradation is also present at the neural scale. Other studies have demonstrated the influence of kinaesthetic feedback in ongoing M1 activity [Gaunt et al., 2013; Herter et al., 2009; Pruszynski et al., 2011; Suminski et al., 2010] suggesting the importance of transcortical feedback pathways for predicting optimal states[Scott, 2012] and for modulating sensory feedback accordingly [Todorov and Jordan, 2002].

Nonbiomimetic approaches, which require the subject to learn to modulate arbitrary but readily discriminable signatures of neural activity [Fetz, 2007; Wolpaw et al., 2002], represent a different approach to BMI which has been explored in paralyzed patients [Birbaumer et al., 2008]. However, it is an open question whether the rate with which subjects can learn to use such systems, or the eventual performance obtained, is also affected by the lack of a functional sensorimotor feedback loop.

Cole [1995] provides a poignant description of the devastating consequences for one patient which sudden loss of large‐diameter afferents had for motor control. Although voluntary movement was regained after much retraining, this required great concentration, and strongly depended on visual feedback. Current cortical prosthetic control resembles Cole's description. Future research is needed to explore alternatives to compensate for such kinaesthetic loss [Weber et al., 2012], which would allow intuitive control of prosthetic devices while maintaining an intact sensorimotor feedback loop.

## CONCLUSION

This study shows that the absence of kinaesthetic feedback from the periphery leads to an impoverished recruitment of the brain neural networks responsible for precise motor control. These results challenge the notion that patients with disconnected neural pathways can still volitionally recall precise motor commands that could be decoded for naturalistic prosthetic control, and suggest that precise naturalistic prosthetic control requires a preserved sensorimotor feedback loop.
